# Detection of the GPI-anchorless prion protein fragment PrP226* in human brain

**DOI:** 10.1186/1471-2377-13-126

**Published:** 2013-09-25

**Authors:** Eva Dvorakova, Tanja Vranac, Olga Janouskova, Maja Černilec, Simon Koren, Anja Lukan, Jana Nováková, Radoslav Matej, Karel Holada, Vladka Čurin Šerbec

**Affiliations:** 1 Institute of Immunology and Microbiology, 1st Faculty of Medicine, Charles University in Prague, Studnickova 7, 128 20 Prague 2, Czech Republic; 2 Department for Production of Diagnostic Reagents and Research, Blood Transfusion Centre of Slovenia, Šlajmerjeva 6, 1000 Ljubljana, Slovenia; 3 Department of Pathology and Molecular Medicine, Thomayer Hospital, Vídeňská 800, 140 59 Prague 4, Czech Republic; 4 Current address: Institute of Macromolecular Chemistry v.v.i., AS CR, Heyrovsky square 2, 162 00 Prague 6, Czech Republic; 5 Current address: Omega, d.o.o., Dolinškova 8, 1000 Ljubljana, Slovenia

**Keywords:** Transmissible spongiform encephalopathies, Creutzfeldt-Jakob disease, GSS, Prion, V5B2, Immunoassay, DELFIA, Anchorless PrP, PrP fragment, Proteinase K

## Abstract

**Background:**

The accumulation of the misfolded forms of cellular prion protein, i.e. prions (PrP^Sc^), in the brain is one of the crucial characteristics of fatal neurodegenerative disorders, called transmissible spongiform encephalopathies (TSEs). Cellular prion protein is normally linked to the cell surface by the glycosylphosphatidylinositol (GPI) anchor. There is accumulating evidence that the GPI-anchorless prion protein may act as an accelerator of formation and propagation of prions. In the TSE affected human brain we have previously discovered a novel GPI-anchorless prion protein fragment, named PrP226*, which ends with the tyrosine 226. This fragment can be labeled specifically by the monoclonal antibody V5B2.

**Methods:**

We developed a DELFIA based assay for quick and sensitive detection of the PrP226* fragment in human brain tissue homogenates. By calculating the ratio between the signals of native (N) and denatured (D) samples applied to the assay we were able to observe significant difference between 24 TSE affected brains and 10 control brains. The presence of PrP226* in brain tissue was confirmed by western blot.

**Results:**

Our results demonstrate that PrP226* is present in small quantities in healthy human brain, whereas in degenerated brain it accumulates in prion aggregates, proportionally to PrP^Sc^. Samples with high D/N ratio generally comprised more proteinase K resistant PrP, while no correlation was found between the quantity of PrP226* and standard classification of Creutzfeldt-Jakob disease (CJD).

**Conclusions:**

In the present study we show that the PrP226* fragment accumulates in prion aggregates and after being released from them by a denaturation procedure, could serve as a proteinase K digestion independent biomarker for human TSEs. The PrP226* assay described in this paper offers a tool to follow and study this unique anchorless PrP fragment in various parts of human brain and possibly also in other tissues and body fluids.

## Background

Transmissible spongiform encephalopathies (TSEs), or prion diseases, are phenotypically heterogeneous group of fatal neurodegenerative disorders of humans and animals. Human prion diseases include Creutzfeldt-Jakob disease (CJD), kuru, Gerstmann-Sträussler-Scheinker disease (GSS) and Fatal familial insomnia. Prion diseases can be sporadic (i.e. spontaneous, 85%), familial (i.e. inherited, 10-15%) or acquired (i.e. transmitted by infection, 2-3%). Prion diseases are characterized by misfolding of the normal cellular prion protein (PrP^C^) into the pathological isoform (PrP^Sc^) [1]. Conversion into abnormal PrP^Sc^ is associated with an increase in β-sheet secondary structure and aggregation of the protein [2,3]. As a result, PrP^Sc^ is insoluble in non-denaturing detergents and partially resistant to protease digestion. Diagnostic methods are mostly based on the treatment of samples with proteinase K (PK) which degrades PrP^C^, while the resistant fragment of pathological prion protein (PrPres) can be detected with anti-PrP antibodies.

Molecular strain typing of human prion diseases has focused mainly on differences in the PrPres size and glycosylation site occupancy in conjunction with the presence of mutations and polymorphisms in the prion protein gene (*PRNP*). Based on differences in gel mobility and N-terminal sequence of the core fragments (PrP27–30) generated by PK digestion, Parchi et al. originally identified two major human PrP^Sc^ types in human prion diseases: type 1 having a relative molecular mass 21 kDa of nonglycosylated PrPres with the primary cleavage site at residue 82 and type 2 having a relative molecular mass 19 kDa with the primary cleavage site at residue 97 [4,5]. The two PrP^Sc^ types in conjunction with *PRNP* gene polymorphism at codon 129, that encodes either methionine (Met) or valine (Val), provided for the first time a molecular basis for disease classification [6]. An alternative classification has been suggested by Collinge et al. [7,8]. In GSS, two different pathological phenotypes are associated either with type 1 PrPres or with a 7-kDa to 8-kDa PrPres fragment [9].

As the research in the field of prion biology advanced, the evidence of the physicochemical heterogeneity of PrP^Sc^ in human and animal prion diseases accumulated [9-13]. The classical typing thus became insufficient for understanding the complex coexistence of distinct structural conformers of PrP^Sc^ in prion diseases formation and progression.

The reports on various truncated forms of the abnormal protein are also in accordance with the structural diversity of the PrP^Sc^. One of the identified PrP fragments is the glycosylphosphatidylinositol (GPI)-anchorless PrP, truncated at the very C terminus [13-15]. Although the role of the GPI anchor in PrP^Sc^ replication and disease propagation remains unclear [16-18], there is increasing evidence on GPI-anchorless PrP, i.e. PrP(ΔGPI), acting as an accelerator of formation and propagation of prions. Heterozygous transgenic mice lacking the sequence for GPI-anchor on one of the PrP alleles, developed clinical signs and died faster than the wild-type mice upon the TSE infection, while their brains appeared to have PrPres generated from both, GPI-anchored and anchorless PrP forms [19]. Stöhr et al. reported recently that mice overexpressing PrPΔGPI developed a spontaneous neurological disease [20]. A similar situation was described in human patients with stop codon mutations at the codons 226 and 227, resulting in the formation of C-terminally truncated PrP, ending either with tyrosine 225 or with tyrosine 226, respectively. Both patients were heterozygous, with the second allele coding for normal PrP, and displayed atypical prion protein amyloidoses [21]. GPI-anchorless PrP was also found on blood cells of patients with paroxysmal nocturnal hemoglobinuria suffering from the clonal defect in GPI synthesis [22,23]. However, in their affected cells the protein seems to be expressed intracellularly, likely in a transmembrane form [23] and patients do not exhibit neurological symptoms.

We previously described development and characteristics of the monoclonal antibody V5B2, raised against human PrP peptide 214–226 [24]. V5B2 specifically recognizes C-terminally truncated fragment of the prion protein that ends with the residue Y226, which was named PrP226* [25]. V5B2 thus represents a unique tool to study localization and behavior of this GPI-anchorless PrP fragment in different biological samples. In this study we developed a V5B2 based sandwich DELFIA immunoassay for detection of the fragment PrP226* in human brain homogenates. Our method is based on comparison of the quantity of PrP226*, measured in native and denatured samples. Its principle is similar to the Conformation-Dependent Immunoassay, reported by Safar et al. [26]. We tested 20 sCJD, 3 fCJD, 1 GSS and 10 non-CJD brain homogenates and we found that the fragment PrP226* accumulates in TSE affected brain, most abundantly in the GSS case. The amount of PrP226* was generally proportional to the amount of PrP^Sc^ and PrPres in prion aggregates.

## Results

### PrP226* C-terminus is degraded by PK

**Figure 1:**
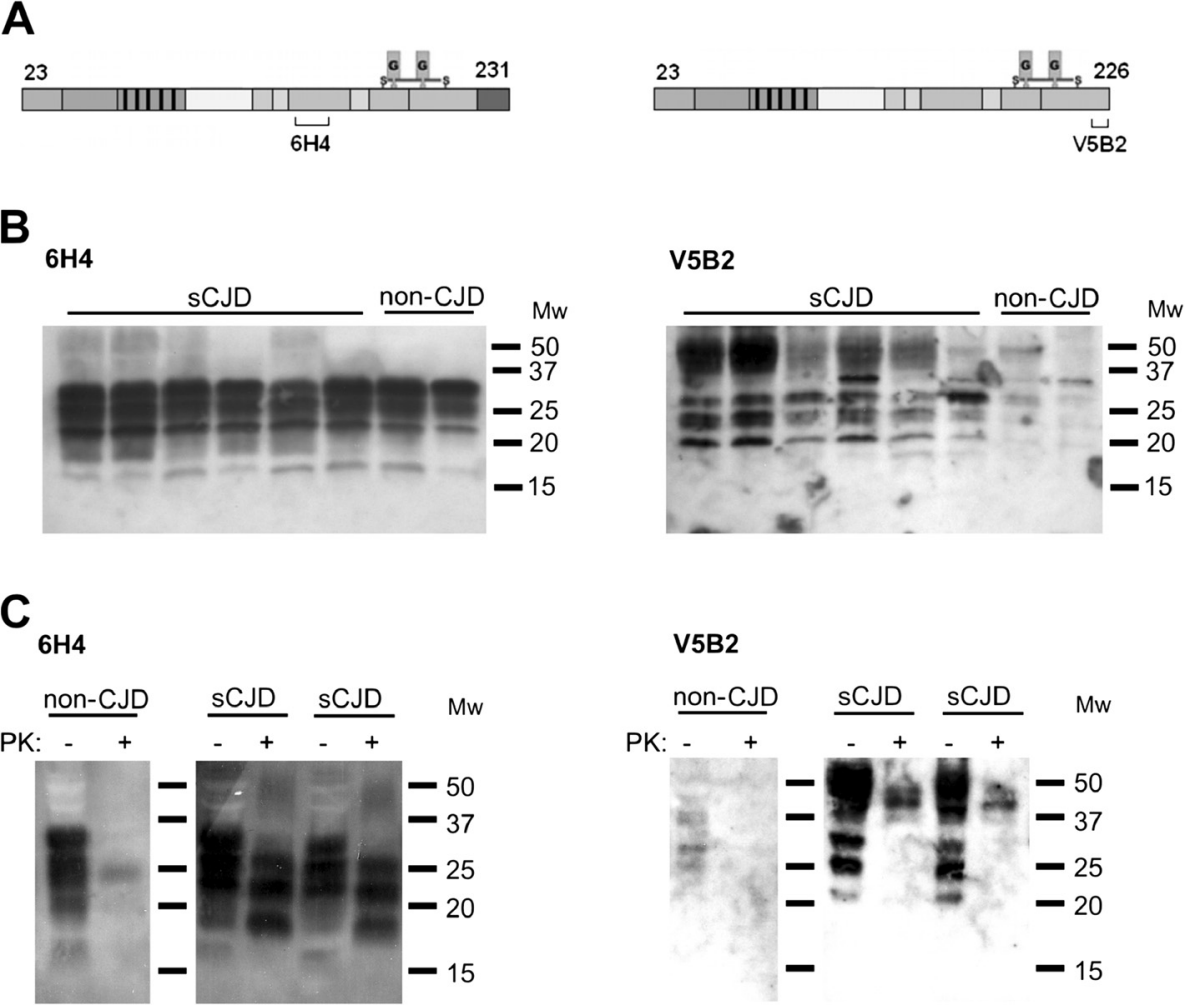
**Western analysis of human brain homogenates. (A)** Epitopes of mAbs 6H4 and V5B2, which were used for immunodetection. **(B)** Analysis of six sCJD and two non-CJD brain homogenates without PK treatment. **(C)** The effect of PK digestion on the immunodetection with mAbs 6H4 and V5B2.

Western blot analyses of sCJD and non-CJD brain homogenates were performed with two anti-PrP mAbs, 6H4, that reacts with the epitope PrP144-152 and V5B2, that reacts with the epitope PrP214-226, with last three amino acids AYY being crucial for binding (Figure 1A). It has been shown before that AYY must be freely exposed; a single amino acid addition to the C-terminus of this epitope completely abolishes V5B2 binding. Therefore V5B2 is specific for the PrP fragment that ends with 226, i.e. PrP226* [25]. PrP226* is barely visible in non-CJD brain, but is present in substantial amounts in human sCJD brain homogenates in all three glycoforms. In contrast, signals of similar intensity were obtained for both sCJD and non-CJD samples when using 6H4 for the immunodetection. PrP226* migrates a few kDa faster than the whole PrP (Figure 1B). PK digestion of samples resulted in PrPres, detected with mAb 6H4, while the V5B2 epitope on PrP226* was largely degraded (Figure 1C).

### Optimization of denaturation conditions for the immunoassay

**Figure 2:**
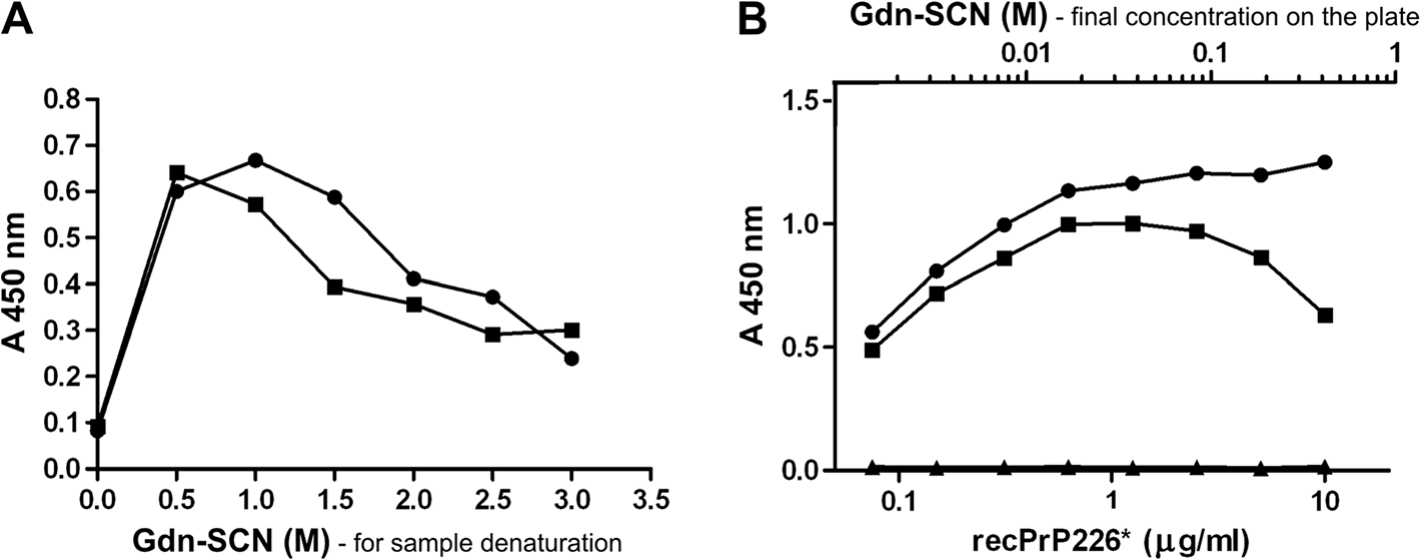
**Determination of the optimal Gdn-SCN concentration for denaturation of samples. (A)** Two 10% sCJD brain homogenates (■ and ●) were treated with increasing concentration of Gdn-SCN (0-3 M). Samples were 10 times diluted and the fragment PrP226* was measured by V5B2/EM20-b sandwich ELISA. **(B)** The effect of Gdn-SCN on the capturing antibody V5B2 was determined using rec PrP226* which was first mixed with 3 M Gdn-SCN 1:1 (v/v), than diluted 5x and further serially diluted prior to loading on a plate (■). Controls were titrated non-denatured recombinant PrP226* (●) and PBS (▲).

We have previously detected PrP226* in PrP^Sc^ deposits in the brain of sCJD patients by immunohistochemistry (IHC) [24]. Therefore, in order to be able to detect the PrP226*, packed in prion aggregates, we set a denaturation protocol for brain homogenates. We first optimized denaturation of samples by Gdn-SCN in a V5B2/EM20-b sandwich ELISA. Two sCJD brain homogenates were treated with increasing concentration of Gdn-SCN at 60°C for 15 min (Figure 2A). When the concentration of Gdn-SCN, to which the samples were exposed, was higher than 1 M, we noticed a substantial decrease of the signal, which could be at least partially attributed to the denaturation of the capturing antibody and thus reducing its binding capacity (Figure 2A). In order to study this effect of Gdn-SCN, we mixed recombinant PrP226* (recPrP226*, at the concentration 10 μg/ml) with 3 M Gdn-SCN, volume ratio 1:1. The mixture was then serially diluted and loaded to the wells of the microtiter plate. The highest signal was obtained at the concentration of 0.075 M Gdn-SCN (Figure 2B). In further experiments, 3 M Gdn-SCN was added to the 10% brain homogenates (vol 1:1). Samples were incubated for 15 min at 60°C and then further diluted 20× to a final concentration of 0.25% brain homogenate in 0.075 M Gdn-SCN.

### PrP226* assay

**Figure 3:**
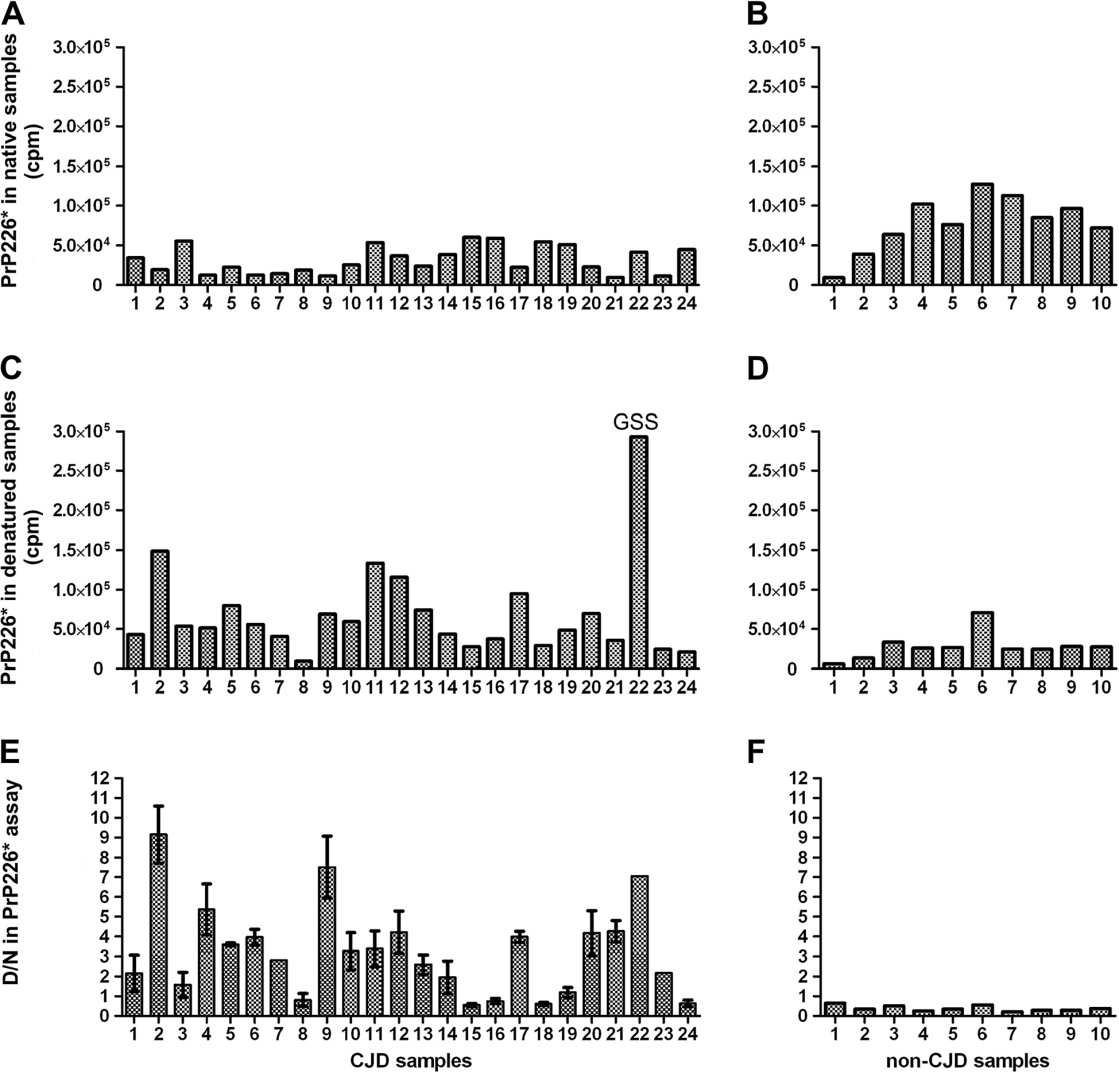
Analyzes of brain homogenate samples with PrP226* assay. 23 CJD and one GSS brain homogenate **(A, C, E)** and 10 non-CJD brain homogenates **(B, D, F)** were analyzed with V5B2/EM20-b DELFIA under native conditions **(A, B)** or after denaturation with 3 M Gdn-SCN for 15 min at 60°C **(C, D)**. Absolute values are represented as counts per minute (cpm), measured by time-resolved fluorescence. The D/N ratio is represented in **(E)** and **(F)**. Error bars were calculated from two independent experiments **(E)**. Absolute measurements of native and denatured samples are presented without error bars, as an example only, as they differed significantly between repeated experiments, due to the sensitivity of DELFIA to slight variations in the protocol.

The determined denaturing and non-denaturing conditions were used to measure the signal of PrP226* in 23 CJD, 1 GSS and 10 non-CJD brain homogenates by sandwich DELFIA. In CJD/GSS samples, the signal at denaturing conditions was usually higher than in non-denaturing conditions (Figure 3A and C), whereas in non-CJD samples, the signal decreased after denaturation (Figure 3B and D). Although this phenomenon was always observed, the measurements of the same samples differed significantly between experiments, due to the sensitivity of DELFIA to slight variations in the protocol. The differentiation between CJD and non-CJD as well as the repeatability of the assay was significantly improved after applying the ratio between the signal of denatured samples and the signal of native samples (D/N, [26]). D/N values of all 10 non-CJD samples were ≤ 0.65 (Figure 3F). CJD/GSS samples formed two groups, the first with D/N ratios between 0.55 and 1.58 (samples 3, 8, 15, 16, 18, 19 and 24), and the second with D/N ratios ≥ 2.0 (all other CJD/GSS samples, Figure 3E).

To investigate whether the low D/N values of the first group of CJD/GSS samples were caused by insufficient homogenization or denaturation of the samples, we repeated the measurements of these samples after additional homogenization by passage through the insulin syringe with 31G needle and by their denaturation at higher temperature (ie. by 1.5 M Gdn-SCN for 10 min at 90°C). However, the treatment did not lead to the change of D/N values of these samples (data not shown).

We proved that D/N correlates with D values alone, i.e. with the concentration of PrP226*, detected after the denaturation of samples (Additional file 1).

### Stability of the fragment PrP226* in human brain homogenates

**Figure 4:**
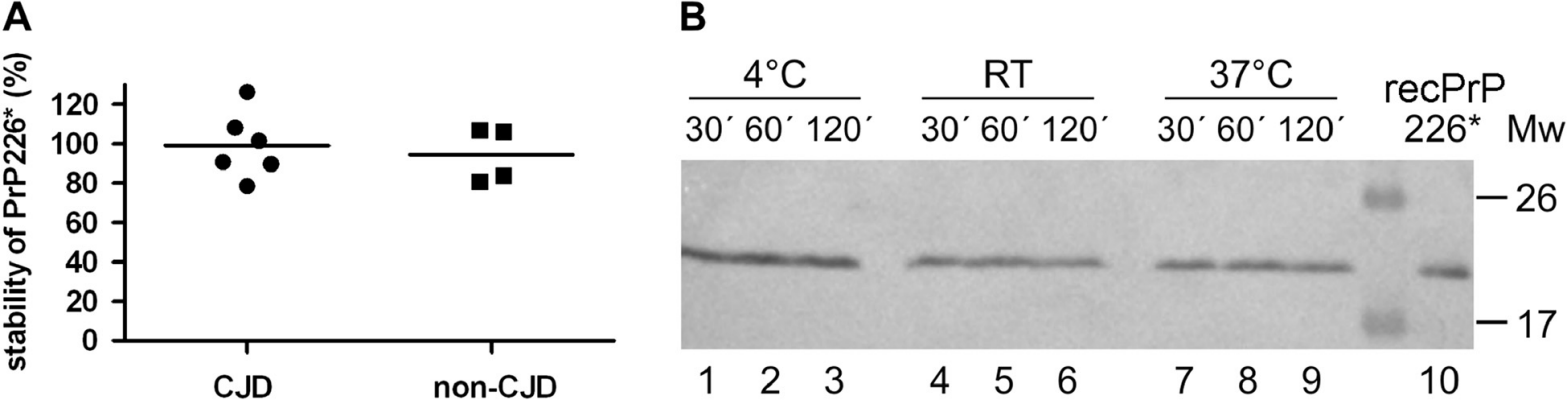
Stability of the PrP226* fragment in brain homogenates. (A) 10% brain homogenates from 6 non-CJD and 6 sCJD patients were split into 2 aliquots, one of each was left at RT overnight and the other was refrozen immediately. Samples were measured on the following day by V5B2/EM20-b sandwich ELISA. Absorbance values of two non-CJD samples were not higher than the background and were therefore not considered. The percentage of the PrP226* signal measured in samples left at RT is calculated relatively to the signal of frozen samples. **(B)** Recombinant human PrP226* (recPrP226*) was spiked into a non-CJD brain homogenate and left for 30 min, 1 h or 2 h at 4°C (lines 1–3), at RT (lines 4–6) or at 37°C (lines 7–9). RecPrP226* was added as a control (line 10). V5B2 (5 μg/ml) was used as detecting antibody.

The stability of PrP226* fragment was determined by V5B2/EM20-b ELISA in the immediately frozen aliquot and the duplicate left overnight at RT. We observed that in average 94.3% (±SD 14.1%) of PrP226* remained in non-CJD and 99.2% (±SD 16.9%) of PrP226* in sCJD brain homogenates (Figure 4A). Additional experiment was performed by spiking recombinant PrP226* into the non-CJD brain homogenate, which was stored at 4°C, at RT or at 37°C. Aliquots, taken at three time points, were examined by western blot. The densitometry analyses showed that, compared to the control recPrP226* (Figure 4B, line 10), the spiked recPrP226* was degraded for 25% at the most, in the aliquot that was left for two hours at RT (Figure 4B).

### Correlation of PrP226* assay with CDI

**Figure 5:**
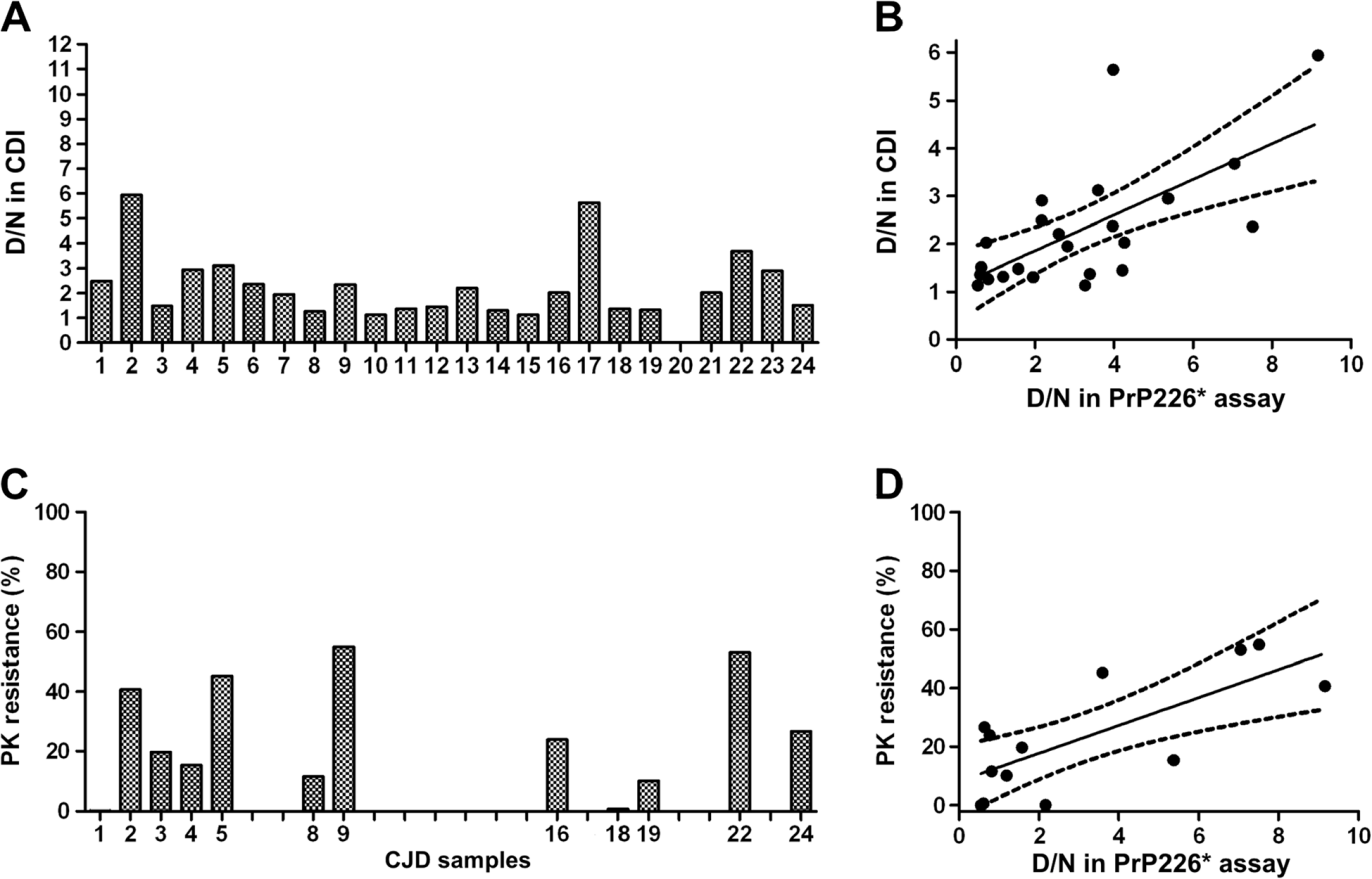
**CDI and PK resistance of PrP**^**Sc **^in CJD samples and the correlation with the PrP226*assay. (A) 23 out of 24 CJD/GSS brain homogenates were analyzed with FH11/3F4-b DELFIA and D/N ratios were calculated. **(B)** Correlation diagram between D/N ratio in PrP226* assay and D/N ratio in CDI. **(C)** 12 out of 24 CJD/GSS brain homogenates were digested with 50 μg/ml PK. Western blots were developed with 3F4, density of bands was evaluated and expressed as a percentage of the resistant PrP compared to the non-digested PrP. **(D)** Correlation diagram between D/N ratio in PrP226* assay and PK resistance.

The results from PrP226* assay were compared to CDI, applied to similar denaturation conditions as were used for the PrP226* assay, with the sole exception that guanidine hydrochloride (Gdn-HCl) was used instead of Gdn-SCN. CDI assay, described by Safar et al. [26], depends on the effect of increasing concentrations of Gdn-HCl to unmask epitopes in PrP^Sc^ that become hidden during the structural rearrangements involved in the formation of PrP^Sc^ and its deposits. In our setting, samples exposed to 4 M Gdn-HCl were detected in a FH11/3F4-b sandwich DELFIA and D/N values were obtained for all CJD/GSS samples (except the sample 20, which was no more available, Figure 5A) and all of the non-CJD samples (not shown). The cut-off D/N value between CJD/GSS and non-CJD measured with CDI was determined to be 1.0. The statistical correlation between the D/N values of CJD/GSS samples measured by V5B2/EM20-b DELFIA and the D/N values of the same samples, measured by CDI by FH11/3F4-b DELFIA (Figure 5B), proved to be highly significant (P value 0.0005 and Pearson correlation factor r = 0.6673) when evaluating all 23 values. These data indicate that the amount of PrP226* in CJD/GSS brain homogenates is proportional to the total amount of misfolded PrP^Sc^ in them.

### PrP226* correlation with PrPres

Next we evaluated the correlation between the PrP226* D/N values and the PK resistance of samples. For this purpose we chose 12 out of 24 CJD/GSS samples which were outstanding due to their very low or very high D/N ratio. Western blot analyses performed with mAb 3F4 showed that standard PK treatment (50 μg/ml, 37°C, 30 min) led to substantial digestion of PrP in the majority of samples, however significant differences in the PrPres amount were detected between the individual samples. Values expressed as percentage of the PrPres signal as to the PrP signal in untreated samples, varied from 0-60% (Figure 5C). When PrP226* D/N ratio was compared to the percentage of PrPres in CJD/GSS samples, we found a correlation for all 12 values (P value 0.0036 and Pearson correlation factor r = 0.7438; Figure 5D). These data indicate that samples with higher PrP226* D/N ratios are in general also more PK resistant.

### PrP226* correlation with standard CJD classifications

**Figure 6:**
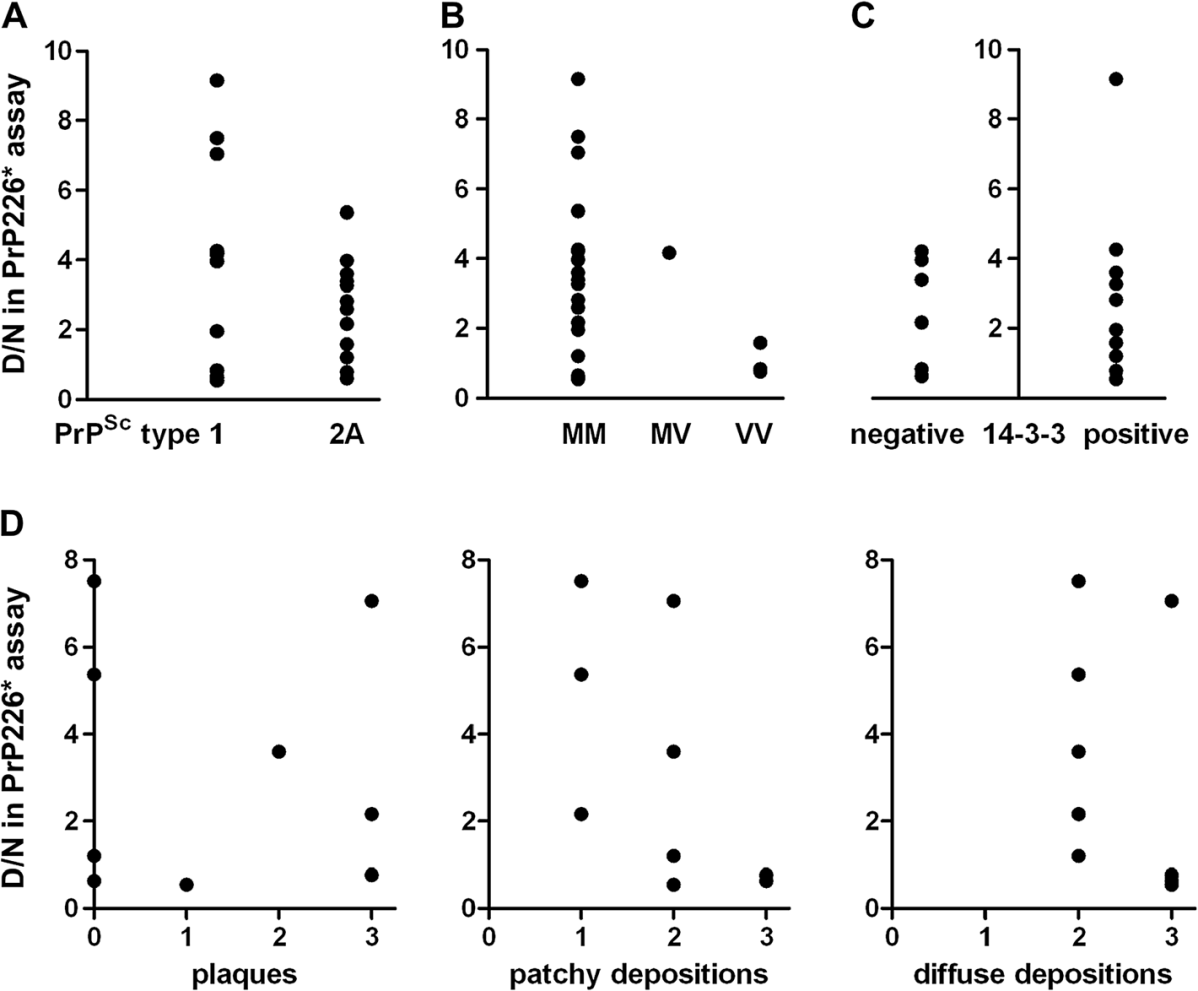
Correlation of D/N ratio in PrP226* assay with standard CJD classifications. The D/N ratio measured in V5B2/EM20-b DELFIA was compared **(A)** to the PrP^Sc^ type, **(B)** to the methionine (M)-valine (V) polymorphism at the codon 129, **(C)** to the presence or absence of the 14-3-3 protein found in CSF and **(D)** to PrP^Sc^ deposition type evaluated by immunohistochemistry with 6H4 after PK cleavage of 9 CJD/GSS samples of frontal cortex. The intensity of prion plaques, patchy/perivacuolar depositions and diffuse/synaptic depositions were evaluated at the scale 0 to 3. In all samples more than one deposition type was found. See also Additional file 2.

To see whether the proportion of PrP226* in deposits (i.e. the D/N ratio in PrP226* assay) correlates with standard CJD sample classifications, D/N ratio measured by PrP226* assay was compared to the PrP^Sc^ type, to the Met/Val polymorphism at the codon 129, to the presence or absence of 14-3-3 protein in the cerebrospinal fluid (CSF) and to the PrP^Sc^ deposition pattern, determined by the immunohistochemical evaluation of CJD/GSS samples. Our results demonstrate that the D/N ratio in PrP226* assay correlates neither with PrP^Sc^ types nor with Met/Val polymorphism at the codon 129 (Figure 6A and B, respectively). We also found no correlation between D/N ratio and the presence of 14-3-3 protein in the CSF (Figure 6C). Finally, IHC was performed on frontal cortex sections of nine chosen samples (samples 1, 4, 5, 8, 15, 16, 19, 22 and 24). The presence of plaques, as well as the morphology of depositions was evaluated but did not correlate with the D/N ratio in PrP226* assay (Figure 6D). For more information on samples, see Additional file 2.

## Discussion

In the present study, we developed a sandwich immunoassay for detecting glycosylphosphatidylinositol (GPI) anchorless PrP fragment ending with amino acid Y226, PrP226* [25]. The method is based on the use of monoclonal antibody V5B2 [24] and enables identification of the PrP226* fragment which is differently accessible in CJD and non-CJD brain tissue (Figure 1B).

The PrP226* assay enables measuring D/N ratio of PrP226* in a brain homogenate, i.e. the signal measured in a denatured sample, divided by the signal measured in the same sample under native conditions. Gdn-SCN-induced denaturation releases previously unavailable PrP226* from prion aggregates, therefore unmasking new V5B2 epitopes. The approach in PrP226* assay is based on similar principles as the Conformation-Dependent Immunoassay [26] and is exploiting chaotropic salts to unmask epitopes buried in prion aggregates. Chaotropic agents like Gdn-SCN, Gdn-HCl or urea disrupt hydrogen bonds responsible for the secondary structure (α-helices and β-sheets) of proteins. Since hydrogen bonding also helps to maintain tertiary and quaternary structures of proteins, denaturation of human brain homogenates induced with Gdn-SCN (or Gdn-HCl), leads to dissociation of prion aggregates as well as to uncoiling of the PrP polypeptide chain.

Different brain tissue samples might need different denaturation conditions for the optimal dissociation of prion aggregates (Figure 2A). However 1.5 M Gdn-SCN was chosen for the PrP226* assay as a compromise to ensure the efficient denaturation of most of the samples. We further optimized the dilution of samples after denaturation to assure that the final concentration of Gdn-SCN present on the microtiter plate affected minimally the binding capacity of V5B2 (Figure 2B). When recombinant PrP226* was used in the assay instead of brain homogenates, a 20% decrease of the signal was observed even when dilution of Gdn-SCN reached 0.075 M. The signal was further dropping rapidly upon the application of denaturant at final concentrations higher than 0.1 M, despite applying higher concentrations of recombinant PrP226* at the same time. The reason for the observed signal decrease could be the structural change of a part of V5B2 paratopes and its epitopes when exposed to denaturant.

The PrP226* assay takes advantage of the monoclonal antibody V5B2, directed against C-terminally truncated fragment of human prion protein that ends with the residue Y226. V5B2 is highly specific for all prion protein fragments ending with the AYY motif. Namely, deprivation of a single tyrosine as well as addition of a single amino acid to tyrosine 226, would completely disrupt the V5B2 binding thus proving that the structure of the epitope at the PrP226* terminus is significantly different from the structure of the corresponding C-terminal parts of fragments PrP225 or PrP227 or of the entire PrP [25]. These findings correspond very well to recent report of Jansen et al. on two patients with stop codon mutations in one allele of the *PRNP* gene, resulting in expression of either PrP225 or PrP226 (i.e. PrP226*, [25]), apart from the normal, GPI-linked PrP. The two patients had strikingly different disease phenotypes, showing that the difference in the exposition of the tyrosine 226 may significantly affect the site of amyloid deposition and the overall phenotype of the prion disease [21].

The PrP226* assay was evaluated using 23 CJD, 1 GSS and 10 non-CJD human brain homogenates. We found that D/N values of PrP226* are lower than 0.7 for non-CJD brain and higher than 1.0 for 80% of CJD/GSS patients’ brain (Figure 3). Moreover, we showed that PrP226* is relatively stable, meaning that only a minor part of it could be ascribed to the *post-mortem* or post–homogenization process of degradation by the released brain proteases (Figure 4A and B). Most of the PrP226* fragment is therefore present in the brain already at the time of death. Our study thus demonstrates that PrP226* accumulates in the prion protein deposits during the course of disease and could therefore serve as a biomarker for human TSEs.

We had the opportunity to also include a GSS sample in our study. This unusual GSS case was caused by a classical P102L mutation, but the manifestation of the disease resembled CJD [27]. The PrP226* assay showed a fivefold higher reaction for the denatured GSS sample, compared to the average signal of other brain homogenates tested (Figure 3C, sample 22), showing a greater abundance of the PrP226* fragment in GSS brain. Interestingly, Jansen et al. also described a GSS-like disease phenotype in a patient expressing PrP226* fragment due to a stop codon mutation on one *PRNP* allele [21].

To further elucidate the reason for D/N ratio differences among the samples, included in our study, we measured the PK resistant fraction of PrP (PrPres) in the samples with either very high or very low D/N (Figure 5C). We found a general correlation between the proportion of PrP226* and PrPres in prion aggregates. However, it is worth stressing that we were able to clearly identify as positive one of the two CJD samples, which were completely degraded by 50 μg/ml PK (Figure 5D, sample 1).

D/N values obtained with the PrP226* assay, correlate with D/N values measured by CDI assay, which implicates that the amount of PrP226* in aggregates is proportional to the amount of total misfolded PrP present in them (Figure 5A and B).

Fragments that might be related to the truncated PrP, PrP226*, have already been described in animal and human TSE affected brain. Stahl et al. showed that 15% of PrP^Sc^ extracted from hamster brain was found to terminate at glycine 228 [14]. Although the site of cleavage of this fragment is different from that of PrP226*, the two fragments might originate from the same mechanism, but differ due to different amino acid sequence between hamster (mouse) and human PrP in the region of protease cleavage site. It is less likely that PrP226* originates in further proteolysis of the PrP228, as PrP226* has never been detected in mice (unpublished data).

Notari et al. identified two GPI-anchorless fragments in human CJD brain that migrate 2–3 kDa faster than PrPres (or PrP27–30) [13]. The fragments, which lack a few amino acids together with the GPI anchor at the very end of the C terminus, have an apparent molecular mass of about 18.5 kDa when associated with PrP27–30 type 1 and of 17 kDa when associated with type 2. Notari et al. also identified a significant heterogeneity in the amount of these fragments among the tested CJD subtypes [13]. When we compared the D/N ratio of PrP226*, measured in 24 CJD/GSS samples, to western blot profiles, to Met/Val polymorphism at the codon 129, to the presence of 14-3-3 protein in CSF or to the type of PrP^Sc^ depositions, we did not find a correlation with any of the mentioned parameters.

In the present study, we also demonstrated that PrP226* can be detected in the brain of some of the patients having no neurological disorders (Figure 3B). It is known that although the majority of the PrP^C^ is bound to the cell membranes via a GPI anchor, a soluble form of PrP^C^ is shed in a normal metabolic process from various cell types, e.g. neurons, splenocytes and platelets [28-30]. Anchorless PrP, i.e. PrP(ΔGPI), has also been identified in the medium of other cultured cells [31,32], in human cerebrospinal fluid and in serum [28,32]. The exact cleavage site on the PrP molecule has not been identified in these studies. Parkin et al. showed that apart from being removed by phospholipase C at serin 231, a significant proportion of PrP^C^ is shed from cell surfaces by the proteolytic action of a zinc metalloprotease which cleaves PrP^C^ very close to the C terminus [15]. Whether the anchorless PrP226* originates in action of zinc metalloproteases and what might be its biological significance in healthy or diseased human brain remains to be determined.

## Conclusions

The PrP226*assay is based on assessing differences between the levels of the soluble form of PrP226* and the PrP226*, packed in prion aggregates. Using this method we were able to show, that this anchorless PrP fragment is a part of prion aggregates in the brain of CJD/GSS patients. We also show that PrP226* can be detected in some non-CJD brains. The role of PrP226* in healthy brain and the importance of its involvement in the pathogenesis of human TSEs remains to be elucidated. However, the accumulation of PrP226* in prion deposits, shown in this study, and its toxicity when overexpressed [21], resembles the recent findings in mice where anchorless PrP was involved in PrP^Sc^ formation [19] and, when overexpressed, also the sole cause of the disease [20,33]. The PrP226* assay, described in this paper, offers a tool to follow and study this anchorless PrP fragment in various parts of human brain and possibly also in other tissues and body fluids.

## Methods

### Antibodies

The following mouse monoclonal antibodies (mAbs) recognizing different epitopes in human PrP were used: V5B2, epitope 214–226 [24]; EM20, epitope in the region 121–231 (EXBIO Praha); FH11, epitope 51–54 (TSE resource centre, Rosslin Institute, Scottland); 3F4, epitope 109–112 (Covance); 6H4, epitope 144–152 (Prionics).

Conjugated antibodies were prepared by covalent labeling of primary antibodies with biotin using EZ-Link® Sulfo-NHS-LC-Biotinylation Kit (Thermo Scientific). The dilutions of the conjugates used in the assays were optimized in titration assays to 0.5 μg/ml for both conjugates, biotinylated EM20 (EM20-b) and biotinylated 3F4 (3F4-b).

### Tissues

The study was carried out on freshly frozen brain tissue samples of one GSS, 20 sCJD and 3 fCJD patients. The study and its protocol were approved by the Central Ethics Committee of Thomayer Hospital and the Institute for Clinical and Experimental Medicine in Prague. In accordance with the guidelines of The Declaration of Helsinki informed consent was obtained either from patient or from relatives and all data were analyzed with respect to patient privacy.

CJD/GSS samples were previously confirmed by the National Reference Laboratory for TSE/CJD in Czech Republic. For negative controls we used freshly frozen brain tissue samples of 10 non-CJD cases without neurological disorders.

PrP^Sc^ typing (according to the fragment size and glycoform ratio) and 14-3-3 determination were performed according to the standard procedures [34].

### Preparation of brain homogenates

sCJD/GSS and non-CJD brain tissue samples were homogenized in 9 volumes of ice-cold lysis buffer (0.5% Sodium deoxycholate, 0.5% Tergitol, 25 mM Tris, pH 7.6) using a HT1000 Potter homogenizer. Brain tissue homogenates (10%, w/v) were not centrifuged, but aliquoted and stored at −80°C.

### V5B2/EM20-b sandwich ELISA

NUNC MaxiSorp microtiter plates were coated with primary antibody V5B2 (1 μg/ml) in carbonate-bicarbonate buffer, pH 9.6 and incubated overnight at 4°C. Plates were than washed with the washing buffer (0.05% Tween/PBS buffer, pH 7.2) and blocked with the blocking buffer (1% BSA in washing buffer). Prepared native and denatured samples were further diluted 20x in the blocking buffer to final 0.25% brain homogenates. 50 μl of samples were loaded and incubated for 90 min at 37°C. Plates were washed and biotinylated antibody EM20-b (0.5 μg/ml), diluted in the blocking buffer, was added for 60 min at 37°C. After washing, avidin-HRP was added (Pierce, 1:5000) for 30 min at 37°C. After a final wash, TMB substrate solution (Pierce) was added. The reaction was stopped after 20 min by addition of 0.1 M H_2_SO_4_ and the absorbance was measured at 450 nm.

### V5B2/EM20-b DELFIA (Dissociation-Enhanced Lanthanide Fluorescent Immunoassay)

DELFIA was performed in the same way as sandwich ELISA until the addition of the biotinylated mAb, with the exception that PerkinElmer Wash Solution was used instead of washing buffer. After this step, plates were washed and further incubated for 30 min at 37°C with streptavidin-Eu (PerkinElmer), diluted to 0.125 μg/ml in Assay Buffer (PerkinElmer). After final washing, Enhancement solution (PerkinElmer) was added. Emission peaks were measured as time-resolved fluorescence at 613 nm from the top of the wells after excitation of the sample at 340 nm, delay 400 μs.

### Stability of the PrP226* fragment

The stability of PrP226*, inherently present in the brain, was measured in fresh brain homogenates of 6 CJD patients and 6 non-CJD individuals. Each homogenate was split in two aliquots, one was left at room temperature over night, while the other was immediately frozen and stored at −80°C until measured. After samples denaturation, following the chosen protocol, the PrP226* was measured by V5B2/EM20-b ELISA, using 5 μg/ml of V5B2 (Figure 4A).

Next, we spiked recombinant PrP226* in 10% non-CJD brain homogenate (without the use of inhibitors) at concentration 1.5 μg/ml. Aliquots were left at 4°C, at RT or at 37°C, respectively. From each aliquot samples were taken at different times (after 30 min, 60 min and 120 min), immediately frozen at −80°C and later loaded simultaneously to the SDS-PAGE gel. Final loading of recPrP226* was 5 ng/well. Western blotting of the gel was performed with V5B2 (5 μg/ml) as detecting antibody, as described below (Figure 4B).

### Optimization of denaturation of samples with guanidine thiocyanate

Two 10% sCJD brain homogenates were mixed with the same amount of stock solution of denaturant to reach increasing final concentrations (from 0 M to 3 M) of guanidine thiocyanate (Gdn-SCN, prepared in 50 mM Tris, pH 8) and incubated at 60°C for 15 min. Samples were than 10 times diluted in 50 mM Tris–HCl, pH 8, and the PrP226* fragment was measured by V5B2/EM20-b ELISA. The plate was coated with 5 μg/ml of V5B2.

To test the denaturing effect of Gdn-SCN on coated antibody, recombinant human PrP226* at 100 μg/ml was divided into 2 aliquots. The first was treated 1:1 (v/v) with 3 M Gdn-SCN at 60°C for 15 min, than diluted 5x and further serially diluted with TBS-T. The second aliquot prepared without denaturant was diluted in the same way.

### PrP226* assay

10% brain homogenates were divided into 2 aliquots, the first was treated 1:1 (v/v) with 3 M Gdn-SCN at 60°C for 15 min. The second aliquot was diluted with the same amount of 50 mM Tris, pH 8, and kept at RT. Both aliquots were further diluted 20x with TBS-T with 1% BSA (final concentration of the brain homogenate was 0.25%) and applied to microtiter plates pre-coated with V5B2 (1 μg/ml). PrP226* fragment was detected by biotinylated EM20 (0.5 μg/ml).

### Conformation dependent immunoassays (CDI)

CDI [26] was performed as a sandwich assay according to Bellon et al. [35], using FH11 (1 μg/ml) as the coating antibody and biotinylated antibody 3F4 (0.5 μg/ml) as detecting antibody, followed by the streptavidin-Eu (PerkinElmer) at concentration 0.125 μg/ml. 10% brain homogenates were denatured with 8 M Gdn-HCl (1:1 v/v) and further diluted to a final concentration of 0.25% before application to the wells. DELFIA was performed as described above.

### Proteinase K digestion of samples

For proteinase K (PK) digestion, brain homogenates were incubated with 50 μg/ml of PK at 37°C and after 30 min boiled in 5x SDS sample buffer (250 mM Tris, pH 6.8, 10% SDS and 50% glycerol) for 5 min.

### Gel electrophoresis and Western blot analysis

Prepared samples were resolved on 12% SDS polyacrylamide gels and proteins on the gels were blotted to nitrocellulose membranes as described previously [36]. For detection, monoclonal antibodies 6H4 (0.2 μg/ml), 3F4 (0.5 μg/ml) or V5B2 (5 μg/ml) were used. For Western blots presented in Figure 1, HRP-conjugated goat anti-mouse IgG + IgM (JacksonImmunoresearch, 0.2 μg/ml) was used, followed by the ECL detection. For all other western blots alkaline phosphatase-conjugated donkey anti-mouse IgG + IgM (JacksonImmunoresearch, 0.2 μg/ml) was used, the reaction was visualized with BCIP substrate and the signal was quantified densitometrically by MiniLumi gel documentation system utilizing GelQuant densitometer software (DNR Bio-Imaging Systems Ltd.).

### Immunohistochemistry

For IHC 5 μm thick sections of formalin-fixed and paraffin-embedded tissue were used. Tissue slices were deparaffinized and washed in TBS. After that they were boiled in citrate buffer (pH 7.6) 3x 5 min in a microwave oven. Endogenous peroxidase was blocked with 0.05 mg of natrium azide and 5 ml of hydrogen peroxide in 50 ml of distilled water. Non-specific positivity was blocked with 150 μl of rabbit serum in 10 ml of TBS for 30 min. Before immunostaining, the sections were sequentially subjected to PK digestion (10 μg/ml, at 25°C for 10 min) and guanidine thiocyanate treatment (2 M, at 25°C for 30 min). The sections were incubated overnight at 4°C with 6H4 monoclonal antibody (1:2000, Prionics) diluted in 5% fetal bovine serum in TBS. Detection of immunostaining was performed using Envision® kit with DAB chromogen. Specimens incubated with secondary antibody only and with nonspecific isotype-matched primary antibodies were used as a control of specificity. Mayer’s haematoxilin was used as a nuclear counterstain.

## Abbreviations

-b: Biotinylated; CDI: Conformation-dependent immunoassay; CJD: Creutzfeldt-Jakob disease; sCJD: Sporadic Creutzfeldt-Jakob disease; CSF: Cerebrospinal fluid; DELFIA: Dissociation-enhanced lanthanide fluorescent immunoassay; D/N: Ratio between assay values for denatured and assay values for native samples; ELISA: Enzyme-linked immunosorbent assay; Gdn-HCl: Guanidine hydrochloride; Gdn-SCN: Guanidine thiocyanate; GPI: Glycosylphosphatidylinositol; GSS: Gerstmann-Sträussler-Scheinker disease; HRP: Horseradish peroxidase; IHC: Immunohistochemistry; mAb: Monoclonal antibody; PK: Proteinase K; PrP: Prion protein; PrPC: Physiological isoform of the prion protein; PrPSc: Pathological isoform of the prion protein; PrP226*: Prion protein’s fragment which ends with the amino acid 226 of the human prion protein sequence; PrPres: Proteinase K resistant core of the prion protein; PRNP: Prion protein gene; SDS-PAGE: Sodium dodecyl sulfate polyacrylamide gel electrophoresis; TBS-T: Mixture of Tris-Buffered Saline and Tween 20; TMB: 3,3′,5,5′-tetramethylbenzidine; TRF: Time resolved fluorescence; TSEs: Transmissible spongiform encephalopathies.

## Competing interests

The authors declare that they have no competing interests.

## Authors’ contributions

ED carried out the experimental work, participated in the design of the study and drafted the manuscript. TV conceived the study, carried out the experimental work and drafted the manuscript. OJ conceived the study, carried out the experimental work and helped to draft the manuscript. MČ carried out the experimental work, participated in the design of the study and helped to draft the manuscript. SK performed western blots of human brain homogenates. AL participated in experimental work. RM carried out immunohistochemical studies. JN performed brain homogenates typing. KH and VČŠ participated in the design and coordination of the study and helped to draft the manuscript. All authors read and approved the final manuscript.

## Pre-publication history

The pre-publication history for this paper can be accessed here:

http://www.biomedcentral.com/1471-2377/13/126/prepub

## Supplementary Material

Additional file 1 Correlation between the D/N ratio and the PrP226* quantity in denatured brain homogenates. The data from this correlation diagram were obtained by V5B2/EM20-b DELFIA. The values of denatured samples (D) are represented in counts per minute (cpm). The line represents the slope of linear regression within the 95% confidence interval. Click here for file

Additional file 2 Characterization of samples by standard CJD classifications and by PrP226* assay. Samples are listed by number together with the result of the sample testing, performed in accordance with standard CJD classification procedures. An empty space in the table indicates that the test was not performed. In the last column the average PrP226* assay results are listed for each sample. Click here for file
