# Supplementary figures and images for: Detection of the GPI-anchorless prion protein fragment PrP226* in human brain
Source: BMC Neurol. 2013 Sep 25;13:126. doi: 10.1186/1471-2377-13-126 (PMC3849060; doi:10.1186/1471-2377-13-126)

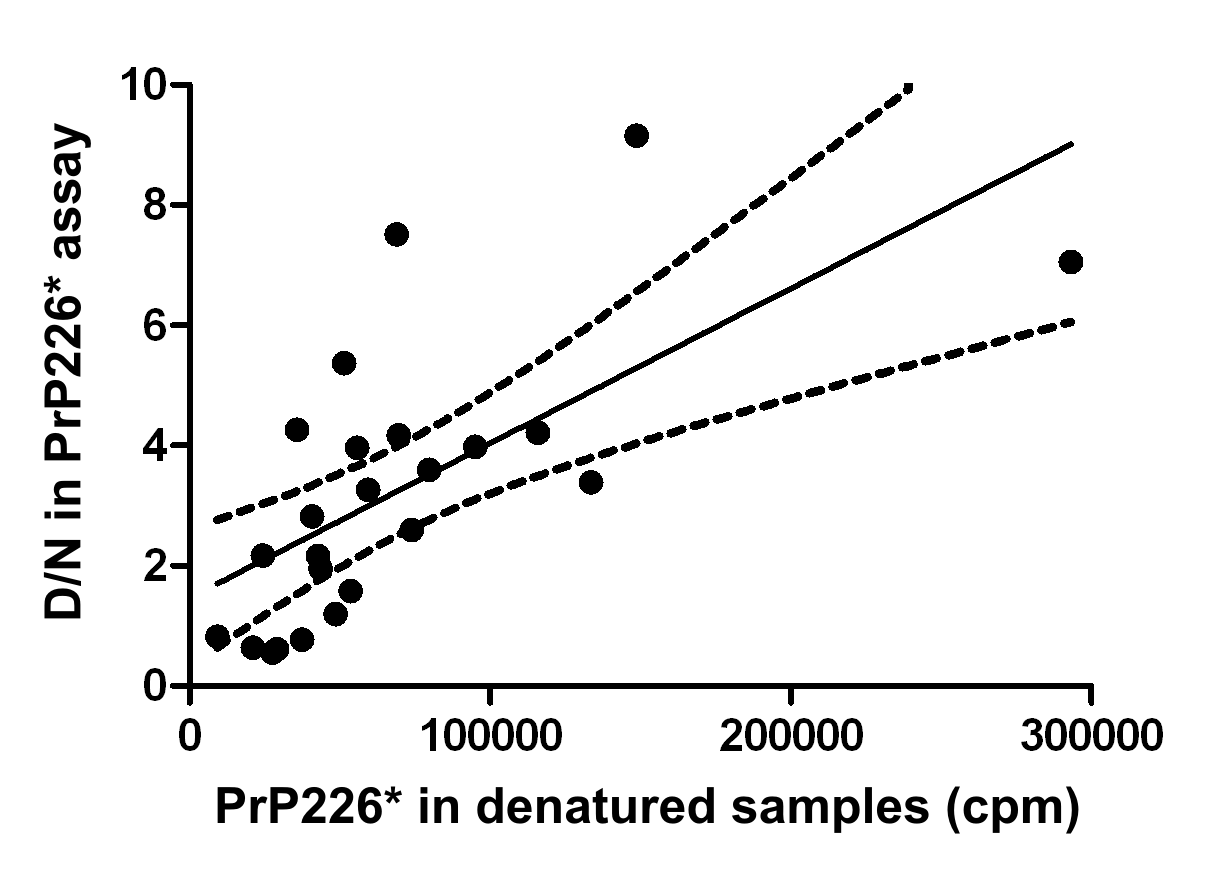

Supplement: Additional file 1 — Correlation between the D/N ratio and the PrP226* quantity in denatured brain homogenates. The data from this correlation diagram were obtained by V5B2/EM20-b DELFIA. The values of denatured samples (D) are represented in counts per minute (cpm). The line represents the slope of linear regression within the 95% confidence interval. [file 1471-2377-13-126-S1.tiff]
