# Supplementary material for: Detection of the GPI-anchorless prion protein fragment PrP226* in human brain
Source: BMC Neurol. 2013 Sep 25;13:126. doi: 10.1186/1471-2377-13-126 (PMC3849060; doi:10.1186/1471-2377-13-126)
Supplement: Additional file 2 — Characterization of samples by standard CJD classifications and by PrP226* assay. Samples are listed by number together with the result of the sample testing, performed in accordance with standard CJD classification procedures. An empty space in the table indicates that the test was not performed. In the last column the average PrP226* assay results are listed for each sample. [file 1471-2377-13-126-S2.docx]

| **Sample** | **14-3-3 protein** | **Codon 129** | **PrP^Sc^ type** | **PrP^Sc^ depozition type** | | | **D/N in PrP226* assay** |
| --- | --- | --- | --- | --- | --- | --- | --- |
|  |  |  |  | plaques | diffuse deposition | patchy deposition |  |
| SPORADIC | | | |  |  |  |  |
| 1 | - | MM | 2A | xxx | xx | x | 2,16 |
| 2 | + | MM | 1 |  |  |  | 9,16 |
| 3 | + | VV | 2A |  |  |  | 1,58 |
| 4 |  | MM | 2A | 0 | xx | x | 5,37 |
| 5 | + | MM | 2A | xx | xx | xx | 3,60 |
| 6 | - | MM | 1 |  |  |  | 3,97 |
| 7 | + | MM | 2A |  |  |  | 2,82 |
| 8 | - | VV | 1 |  |  |  | 0,82 |
| 9 |  | MM | 1 | 0 | xx | x | 7,51 |
| 10 | + | MM | 2A |  |  |  | 3,27 |
| 11 | - | MM | 2A |  |  |  | 3,39 |
| 12 | - | MM | 1 |  |  |  | 4,22 |
| 13 |  | MM | 2A |  |  |  | 2,60 |
| 14 | + | MM | 1 |  |  |  | 1,96 |
| 15 | + | MM | 1 | x | xxx | xx | 0,55 |
| 16 | + | VV | 2A | xxx | xxx | xxx | 0,77 |
| 17 |  | MM | 2A |  |  |  | 3,98 |
| 18 |  | MM | 2A |  |  |  | 0,62 |
| 19 | + | MM | 2A | 0 | xx | xx | 1,20 |
| 20 |  | MV | 1 |  |  |  | 4,17 |
| FAMILIAL | | | |  |  |  |  |
| 21 | + | MM | 1 |  |  |  | 4,27 |
| 22 **GSS** |  | MM | 1 | xxx | xxx | xx | 7,05 |
| 23 | - | MM | 2A |  |  |  | 2,17 |
| 24 | - | MM | 1 | 0 | xxx | xxx | 0,64 |
